# Prior Infection of Chickens with H1N1 or H1N2 Avian Influenza Elicits Partial Heterologous Protection against Highly Pathogenic H5N1

**DOI:** 10.1371/journal.pone.0051933

**Published:** 2012-12-11

**Authors:** Charles Nfon, Yohannes Berhane, John Pasick, Carissa Embury-Hyatt, Gary Kobinger, Darwyn Kobasa, Shawn Babiuk

**Affiliations:** 1 National Centre for Foreign Animal Disease, Canadian Food Inspection Agency, Winnipeg, Manitoba, Canada; 2 National Microbiology Laboratory, Public Health Agency of Canada, Winnipeg, Manitoba, Canada; 3 Department of Animal Science, University of Manitoba, Winnipeg, Manitoba, Canada; 4 Department of Medical Microbiology, University of Manitoba, Winnipeg, Manitoba, Canada; 5 Department of Immunology, University of Manitoba, Winnipeg, Manitoba, Canada; The Ohio State University, United States of America

## Abstract

There is a critical need to have vaccines that can protect against emerging pandemic influenza viruses. Commonly used influenza vaccines are killed whole virus that protect against homologous and not heterologous virus. Using chickens we have explored the possibility of using live low pathogenic avian influenza (LPAI) A/goose/AB/223/2005 H1N1 or A/WBS/MB/325/2006 H1N2 to induce immunity against heterologous highly pathogenic avian influenza (HPAI) A/chicken/Vietnam/14/2005 H5N1. H1N1 and H1N2 replicated in chickens but did not cause clinical disease. Following infection, chickens developed nucleoprotein and H1 specific antibodies, and reduced H5N1 plaque size *in vitro* in the absence of H5 neutralizing antibodies at 21 days post infection (DPI). In addition, heterologous cell mediated immunity (CMI) was demonstrated by antigen-specific proliferation and IFN-γ secretion in PBMCs re-stimulated with H5N1 antigen. Following H5N1 challenge of both pre-infected and naïve controls chickens housed together, all naïve chickens developed acute disease and died while H1N1 or H1N2 pre-infected chickens had reduced clinical disease and 70–80% survived. H1N1 or H1N2 pre-infected chickens were also challenged with H5N1 and naïve chickens placed in the same room one day later. All pre-infected birds were protected from H5N1 challenge but shed infectious virus to naïve contact chickens. However, disease onset, severity and mortality was reduced and delayed in the naïve contacts compared to directly inoculated naïve controls. These results indicate that prior infection with LPAI virus can generate heterologous protection against HPAI H5N1 in the absence of specific H5 antibody.

## Introduction

Influenza A viruses can infect a variety of animal species including birds, swine and humans. Highly pathogenic avian influenza continues to cause economic losses to the poultry industry worldwide with outbreaks of H5N2 and H7N3 in North America [1], [2], [3] as well as outbreaks of H5N1 originating in Hong Kong [4], [5] spreading through out Asia and into Africa and Europe. These Eurasian H5N1 are zoonotic and can cause serious disease leading to death in humans [6] and are feared of causing the next influenza pandemic [7]. The demonstration that H5N1 through a combination of mutations can transmit between ferrets has further raised alarms that H5N1 could cause the next influenza pandemic [8], [9]. Influenza viruses are segmented negative-sense single stranded RNA viruses and can undergo genetic drift when the individual genes change slowly through mutation over time or genetic shift where entire gene segments can be exchanged between different influenza viruses. The reservoir for avian influenza are wild birds where hemagglutinin (HA) (H1–H16) and neuraminidase (NA) (N1–N9) subtypes circulate [10], [11]. Recently an H17 subtype has been discovered in bats [12]. In birds, low pathogenic avian influenza (LPAI) viruses replicate but do not cause severe clinical disease, however LPAI can result in a drop in egg production even when no clinical signs are observed. However, highly pathogenic avian influenza (HPAI) can evolve from some H5 and H7 subtype viruses by the acquisition of a polybasic amino acid motif at the HA_0_ cleavage site. Highly pathogenic avian influenza causes severe clinical disease and death in poultry [1].

There is a currently an unmet need to have a vaccine that can protect against newly emerging influenza viruses prior to knowing their subtype to develop a vaccine. Although currently used conventional influenza vaccines are generally effective in protecting animals and humans if used properly, they are not ideal since new vaccines need to be matched and generated against currently circulating influenza viruses. This lag time in vaccine generation was demonstrated by the H1N1 2009 pandemic where a vaccine was not available at the start of the pandemic [13]. Therefore the development of universal influenza vaccines able to protect against an unknown newly emerging pandemic influenza virus is critical. To generate a universal vaccine the correlates of immune protection against influenza would be valuable to aid development. Currently, influenza neutralizing antibodies are one known correlate of immunity. However, a universal vaccine eliciting neutralizing antibodies against multiple influenza virus subtypes is currently not feasible because the generation of escape mutants can occur through genetic drift [14]. Killed influenza vaccines must be closely matched with the HA subtype to be effective and even small changes result in the vaccine losing effectiveness [15]. It is possible to generate cell mediated immunity to protect against different influenza subtypes, using a variety of approaches. These include DNA vaccines [16], vector based vaccines [17] and attenuated influenza viruses [18]. Heterologous immunity has been demonstrated to influence influenza virus infection [19]. Furthermore, the role of natural infection with influenza viruses in generating heterologous immunity against HPAI H5N1 influenza has been evaluated in various animal models such as ferrets [20], pigs [21], Canada geese [22], wood ducks [23], mallard ducks [24], swans [25] and chickens [26]. These publications demonstrate that previous infection with several different live influenza viruses can either protect or influence the outcome of HPAI influenza virus infection in a wide variety of animal species. Hence, prior infection with a heterologous influenza virus may offer potential protection against pandemic influenza viruses. Evaluating heterologous immunity generated by prior infection with influenza virus may lead to improved vaccination strategies. Chickens were chosen to evaluate heterologous immunity since they are highly susceptible to HPAI, and HPAI influenza is transmissible between chickens. Hence, heterologous protection of chickens may provide insight into heterologous protection of other species. To address the role of heterologous immunity following natural infection we used LPAI A/goose/AB/223/2005 H1N1 or A/WBS/MB/325/2006 H1N2 virus to infect chickens prior to challenge with A/chicken/Vietnam/14/2005 H5N1. In addition, the transmission of H5N1 from chickens previously infected with LPAI prior to H5N1 challenge to naïve contact chickens was assessed.

## Materials and Methods

### Ethics Statement

All animal work was carried out in compliance with Canadian Council on Animal Care guidelines and was approved by the Animal Care Committee at the Canadian Science Centre for Animal and Human Health.

### Viruses

A/goose/AB/223/2005 H1N1 was isolated in 2005 from a wild goose in Alberta Canada. A/WBS/MB/325/2006 H1N2 was isolated in from a wild bird in Manitoba Canada. Both these viruses were grown and titrated in 9–10 days old embryonated chicken eggs. A/chicken/Vietnam/14/2005 H5N1 (H5N1) used in this study had an intravenous pathogenicity index (IVPI) score of 2.9. Propagation and titration of this H5N1 virus was done in Japanese quail fibrosarcoma (QT-35) cells. The sequence similarity at the amino acid level of N1 from H5N1 was 87% similar to N1 from H1N1 and 43% similar to N2 from H1N2. In addition, the N1 from the H5N1 had a 20 amino acid deletion in the stalk region.

### Infection of chickens with H1N1 and H1N2

Specific pathogen free (SPF) chickens were obtained at 40 days of age from the Ottawa CFIA Fallowfield laboratory. The birds were floor housed in heated enhanced BSL3 animal cubicles and allowed 1 week of acclimatization before the start of experiments. Each chicken was inoculated with 10^5^ plaque forming units (pfu) of H1N1 or H1N2 in 1 ml sterile PBS via the cloaca, trachea, nares and eyes. Chickens were monitored twice daily and clinical signs scored as 0, 1, 2 or 3 for normal, mildly sick, sick, and very sick (moribund) respectively. Cloacal and oropharyngeal swabs were collected from each chicken on day 0 (prior to challenge) and at predetermined time points post challenge. Blood for serum separation was collected from the wing vein of anaesthetized chickens on day 0 and 21 days post infection (DPI). Swabs and sera were stored at −70°C.

### Heterologous challenge and evaluation of heterologous cross-protection

Chickens pre-infected with LPAI H1N1 or LPAI H1N2 at 21 DPI as well as aged matched control chickens were used for the H5N1 challenge. The H5N1 challenge groups were 1) 9 LPAI H1N1 infected plus 5 uninfected control chickens co-housed in the same cubicle, 2) 9 LPAI H1N1 infected chickens with 5 non infected contact controls added to the cubicle the day after the challenge, 3) 10 LPAI H1N2 infected chickens with 5 uninfected control chickens co-housed in the same cubicle, and 4) 10 LPAI H1N2 infected chickens with 5 non infected contact controls added to the room the day after the challenge. The H5N1 challenge was performed using 10^5^ pfu of H5N1 delivered by the intranasal, oral and ocular routes. Clinical signs were monitored and scored as previously described. For animal welfare reasons, moribund chickens were humanely euthanized and assigned a clinical score of 3. The number of dead and/or euthanized birds was recorded. Cloacal and oropharyngeal swabs were collected from each chicken prior to and at predetermined days post challenge (DPC). Blood for serum separation was also collected from the wing vein of surviving chickens at 21 DPC.

### RNA extraction and quantitative real-time reverse transcriptase polymerase chain reaction (qRT-PCR)

Total RNA was extracted from 0.5 mL of clarified oral and cloacal swab specimens using the RNeasy Mini kit (Qiagen, Mississauga, Ontario, Canada) according to the manufacturer's protocol. To semi-quantify the amount of virus nucleic acid in each swab specimen, a semi-quantitative real-time RT-PCR specific for the M1 gene of influenza A was performed as previously described [27]. Standard curves were generated for each run using serial dilutions of full length *in vitro* transcribed influenza A Matrix gene. The nucleic acid copy number in each specimen was extrapolated from the standard curve. In addition, qRT-PCR specific for H5 was performed to confirm the post challenge shedding was H5N1.

### Serology

All serum samples were heat-inactivated in a water bath for 30 minutes at 56°C.

Hemagglutination-inhibition (HI) assay was performed according to the WHO manual on animal influenza diagnosis and surveillance protocol [28]. Briefly, 4 HA units of virus were added to equal volumes of 2-fold serially diluted sera and incubated at room temperature for 30 minutes. This was followed by the addition of a 0.5% (V/V) suspension of chicken red blood cells (CRBC). The highest dilution of serum which completely inhibited the agglutination of CRBC was determined, the reciprocal of which was considered the HI titre for that serum specimen.

The neuraminidase inhibition (NI) assay was performed as previously described [29], using binary ethylenimine (BEI)-inactivated whole LPAI H1N1 virus antigen. The antigen was first titrated to determine the optimum antigen dilution for use in NI assay. The presence of anti-NA antibodies in serum and the NI titre were then detected by adding the optimal antigen concentration to serial 2-fold dilutions of sera. The highest dilution of anti-serum to inhibit NA activity was considered the NI titre.

Virus neutralisation assay was also performed according to the WHO manual on animal influenza diagnosis and surveillance protocol [28], with minor modifications. Briefly, equal volumes of 100 pfu of influenza virus and serial 2 fold dilutions of sera were mixed and incubated at 37°C. After 1 hour, 100 uL of serum/virus mixture was transferred to corresponding wells of 96-well plate with confluent MDCK cells (ATTC CCL-34) and incubated at 37°C for 1 hour. The serum/virus mixture was then replaced with fresh culture medium and plates incubated at 37°C for 3–4 days. Plates were examined for cytopathic effect (CPE) and the reciprocal of the highest serum dilution to completely neutralize AIV growth in at least 2 out of 4 wells was considered the VN titre for that serum sample. Serological cross-reactivity between H1N1 or H1N2 and the HPAI H5N1 was assessed by testing the virus in combination with sera from LPAI H1N1 or H1N2-infected chickens in both HI and VN assays. The competitive ELISA for detecting antibodies against influenza A nucleoprotein (NP) was performed as previously described [30], using a baculovirus-expressed recombinant influenza A NP antigen.

Plaque size reduction assay was performed following a published protocol [31] with some modifications. Briefly, 50 pfu of H5N2 in 500 μL AMEM was added into each well containing a confluent monolayer of MDCK cells in a 12-well plate. Virus back titration and cell control wells were also included. After a 1 hour incubation at 37°C, the virus inoculum was removed from all wells and 1.5 mL of DPI 21 serum from LPAI H1N1-infected chickens diluted 1/10, 1/50 and 1/100 in an overlay of 1.5% carboxymethylcellulose, 2% FBS in DMEM (CMC overlay) was added to duplicate wells of H5N2 infected cells. Overlay lacking chicken serum was added to the virus back titration and the cell control wells. After 96 hours at 37°C, the cells were fixed in 10% phosphate buffered formalin and immunostained using anti-influenza A NP monoclonal antibody. Plaques were visualized under an Olympus microscope and plaque diameter determined using a computer based cell Sens Imaging software version 1.4.1 (Olympus Corporation). The average plaque size of at least 20 plaques per serum dilution was calculated.

### Cell mediated immunity

Peripheral blood mononuclear cells (PBMC) were purified from heparinized blood collected from 4 H1N1 infected and 2 uninfected control chickens, using Ficoll-Paque™ Plus (GE Healthcare Bio-Sciences AB, Uppsala, Sweden) following a previously described protocol [32]. Purified PBMC were resuspended in RPMI-1640 supplemented with 2 mM L-glutamine, 100 U/ml penicillin, 100 ug/ml streptomycin and 10% chicken serum (culture medium). To detect cell mediated immune (CMI) responses, PBMC were stimulated with H5N1 antigen and IFN-γ secretion and cell proliferation measured. PBMC were resuspended in culture medium at 1×10^7^ cells/ml and 100 ul (1×10^6^ cells) transferred to individual wells of a 96-well cell culture plate. Duplicate wells were stimulated with 100 ug/ml of purified BEI-inactivated H5N1 antigen to give a final volume of 200 ul/well. Cultures were incubated for 48 hours at 37°C and supernatants harvested after removing the cells by centrifugation. IFN-γ concentrations in supernatants were measured by ELISA using Chicken IFN-γ CytoSet™ (Invitrogen, Camarillo, CA, USA) according to the manufacturer's protocol. Antigen-driven PBMC proliferation was assayed by the carboxyfluorescein diacetate, succinyl ester (CFSE) dilution method [32]. PBMC were first labelled with CFSE according to manufacturer's protocol (Molecular Probe™, Eugene, OR, USA), then resuspended in culture medium and 1×10^6^ cells in 100 ul transferred to individual wells of a 96-well cell culture plate. Duplicate wells were stimulated as described above. Cultures were incubated at 37°C for 5 days, PBMC from each well resuspended by pipetting up and down and then transferred to FACS tubes. A 2 laser Flow cytometer (Beckman Coulter, Mississauga, ON, Canada) was used to determine the percentage of proliferated cells and the data analyzed with CXP Software (Beckman Coulter). The percent proliferation value for antigen-stimulated cells was divided by that for unstimulated controls to obtain the stimulation index (SI).

### Statistics

Data from multiple time points was analyzed by 2-way ANOVA and Bonferroni multiple comparisons post test, using GraphPad Prism version 5. Differences between pairs of data collected at a single time point were analyzed by student t-test. Any p<0.05 was considered statistically significant.

## Results

### Chickens infected with A/goose/AB/223/2005 H1N1 or A/WBS/MB/325/2006 H1N2 develop no clinical disease despite virus replication and shedding

**Figure 1:**
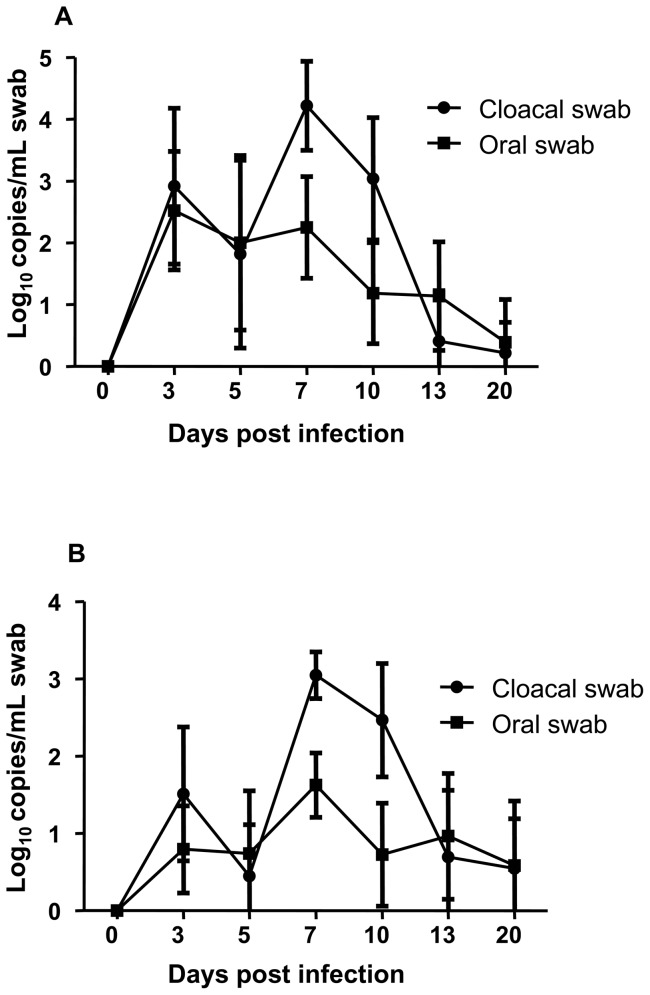
Influenza viral genome copies in oral and cloacal swabs following H1N1 and H1N2 infection. Oral and cloacal swabs were collected prior to and following H1N1 (A) and H1N2 (B) infection. Viral RNA genome copies were determined by quantitative real time RT-PCR assay. Error bars represent the standard deviation of mean of 18 chickens for H1N1 and 20 chickens for H1N2.

All chickens infected with H1N1 or H1N2 avian influenza shed virus beginning on DPI 3 as detected by quantitative real-time RT-PCR (Figure 1). Virus shedding peaked on DPI 7 and was undetectable in most chickens at DPI 20. There was significantly more virus in cloacal than in oral swabs at DPI 7 and 10 (p<0.05) for both H1N1 and H1N2. However, despite the replication and shedding of virus, all H1N1 and H1N2 infected chickens showed no signs of disease throughout the 21 days of observation. The kinetics and duration of shedding was very similar for both H1N1 and H1N2 infections.

### Chickens serconverted following A/goose/AB/223/2005 H1N1 and A/WBS/MB/325/2006 H1N2 infection

**Figure 2:**
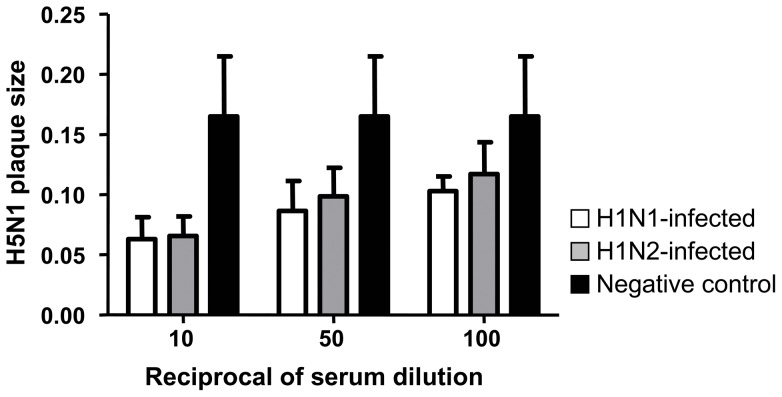
Antibodies from H1N1 and H1N2 infected chickens reduce H5N1 plaque size. MDCK cells were infected with H5N1 in the presence of sera from H1N1-infected (open histograms), H1N2-infected (grey histograms) or naïve (black histograms) chicken and plaque size (mm) determined after 3 days. Data represents mean and error bars are standard deviation for sera from at least 5 chickens per group.

Serum samples collected from all chickens prior to H1N1 or H1N2 infection had no HI or VN antibodies specific for H1N1 or H1N2. However, at 21 DPI all H1N1 infected chickens elicited H1-specific HI (338 mean titre with 239 standard deviation) and VN (840 mean titre with 436 standard deviation) antibodies and all H1N2 infected chickens had H1-specific HI (188 mean titre with 181 standard deviation) and VN (920 mean titre with 399 standard deviation) antibodies. In addition, all the serum samples at 21 DPI from H1N1 or H1N2 infected chickens tested positive for influenza A NP antibodies with greater than 95% inhibition in the cELISA confirming influenza infection. All H1N1 infected chickens at 21 DPI developed N1-specific antibodies with the N1 neuraminidase inhibition assay (212 mean titre with 74 standard deviation). Chickens infected with the N2 subtype did not react in the N1 neuraminidase inhibition assay. In contrast, no heterologous antibody activity against H5N1 was detected in sera from H1N1 or H1N2 infected chickens using VN assays. Although there were no neutralizing antibodies specific for H5N1 in chicken sera at 21 DPI following either H1N1 or H1N2 infection, these antibodies were able to significantly decrease H5N1 virus plaque size compared to negative sera (Figure 2). There was no significant difference in H5N1 plaque size when sera from H1N1 and H1N2 infected chickens were compared against each other. However, when compared against negative control sera, antibodies from H1N1 infected chickens significantly reduced H5N1 plaque size at all dilutions tested (p<0.05). Similarly, antibodies from H1N2-infected chickens significantly reduced H5N1 plaque size at 1/10 and 1/50 dilutions (p<0.05) compared to negative control sera.

### Infection of chickens with A/goose/AB/223/2005 H1N1 induces heterologous cell mediated immune responses to H5N1

**Figure 3:**
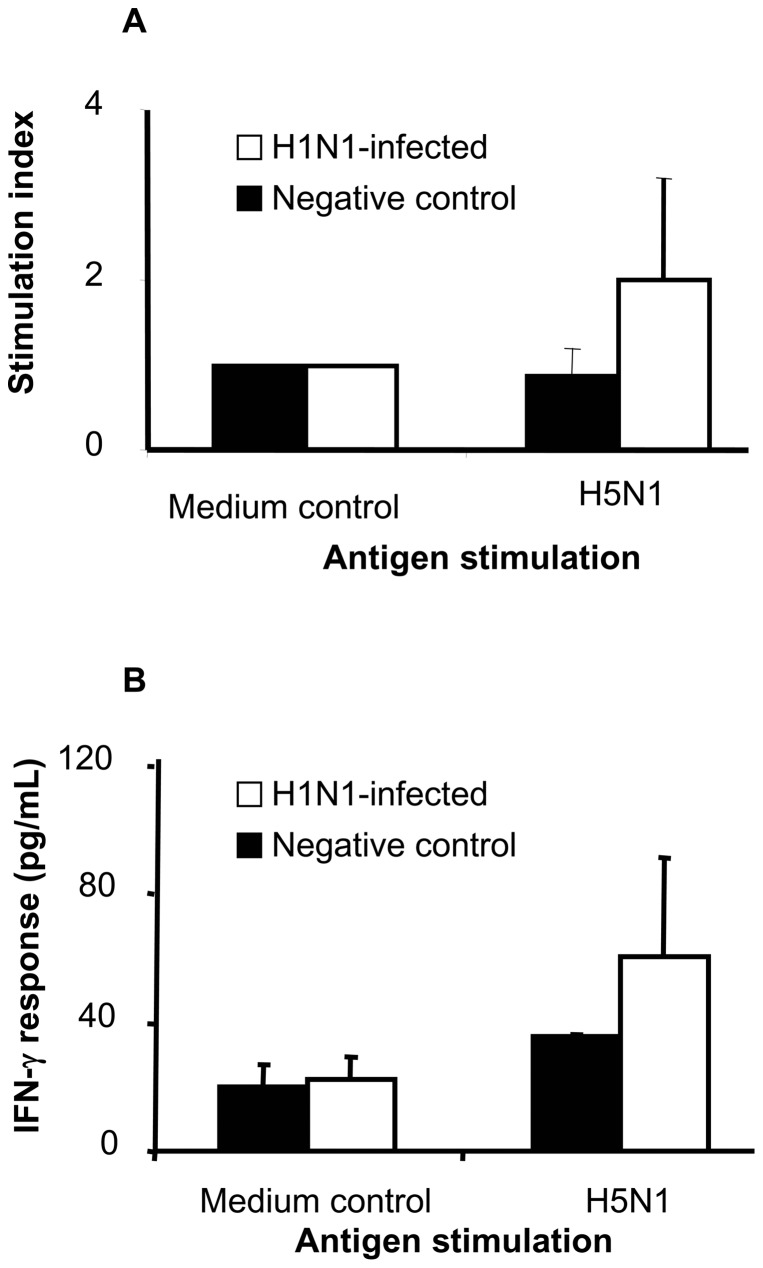
Heterologous cell mediated immune response following H1N1 infection. PBMCs were obtained following infection with H1N1 and then re-stimulated with H5N1 antigen. Cell proliferation (A) and IFN-γ secretion (B) were measured. The bar represents the mean with standard deviation of 4 H1N1-infected and 2 control chickens.

Heterologous cell mediated responses were assessed by antigen-induced IFN-γ secretion and PBMC proliferation. PBMC from LPAI H1N1-infected chickens proliferated more than PBMC from uninfected controls following stimulation with H5N1 antigen (Figure 3A). Similarly, stimulation of PBMC from LPAI H1N1-infected chickens with inactivated H5N1 antigen induced close to 50% higher IFN-γ secretion compared to PBMC from uninfected controls (Figure 3B). The higher IFN-γ and proliferative response in LPAI H1N1-infected chickens was indicative of a heterologous recall response.

### Prior infection with LPAI protects chickens against HPAI H5N1

**Figure 4:**
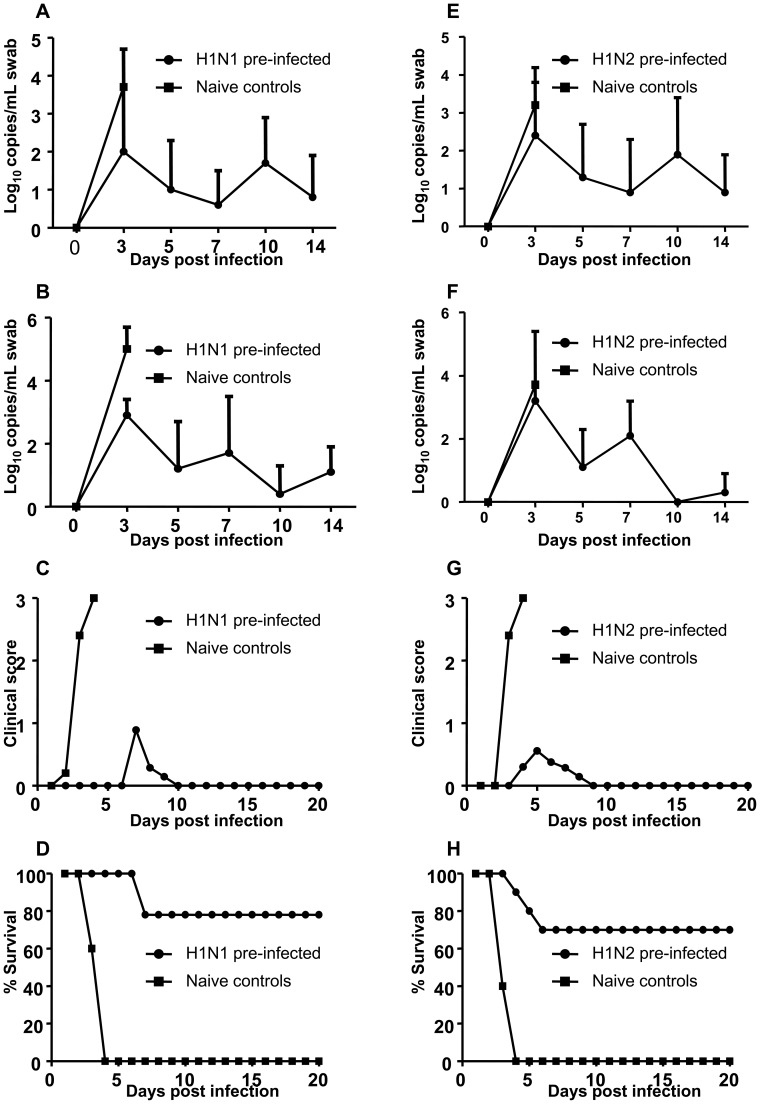
Virus shedding, clinical scores and survival following H5N1 challenge of both pre-infected and naïve chickens. Chickens previously infected with H1N1 (A–D) or H1N2 (E–H) as well as naïve control chickens were challenged with H5N1. Following H5N1 challenge virus shedding determined by quantitative real time RT-PCR in oral swabs (A and E) and cloacal swabs (B and F); clinical score (C and G) and survival of chickens (D and H) were monitored.

Following H5N1 challenge, oral and cloacal swabs were collected on days 3, 5, 7, 10 and 14 and evaluated for influenza viral RNA using qRT-PCR. When A/goose/AB/223/2005 H1N1 pre-infected and control chickens were co-housed and all challenged with H5N1, the control chickens shed higher levels of virus in both oral and cloacal swabs at 3 DPC (Figure 4A and B). The difference in virus shedding between the 2 groups was statistically significant for 3 DPC cloacal swabs (p<0.05). In H1N1 pre-infected chickens, H5N1 shedding peaked at 3 DPC and decreased with time. Control chickens started to develop clinical signs of H5N1 disease at 2 DPC (Figure 4C). In contrast, the majority of chickens pre-infected with H1N1 did not develop clinical signs of disease until 7 DPC when 3 chickens developed clinical signs. The difference in clinical score between the 2 groups reached statistical significance on 3 and 4 DPC (p<0.05). All control chickens were dead by 4 DPC but all H1N1 pre-infected chickens survived H5N1 challenge until 8 DPC when 2 of 9 died (Figure 4D).

Similarly, when A/WBS/MB/325/2006 H1N2 pre-infected and control chickens were co-housed and all challenged with H5N1, the control chickens shed slightly higher levels of virus in both oral and cloacal swabs at 3 DPC (Figure 4E and 4F). Likewise, control chickens started to show clinical signs of H5N1disease at 2 DPC (Figure 4G) but only 3 of 10 H1N2 pre-infected chickens developed clinical signs starting at 4 DPC (Figure 4G). The difference in clinical score between the 2 groups reached statistical significance on 3 and 4 DPC (p<0.05). All control chickens were dead by 4 DPC while only 3 of 10 H1N2 pre-infected chickens died of H5N1 by 7 DPC (Figure 4H).

### Prior infection with LPAI does not prevent H5N1 transmission to contact control chickens following challenge

**Figure 5:**
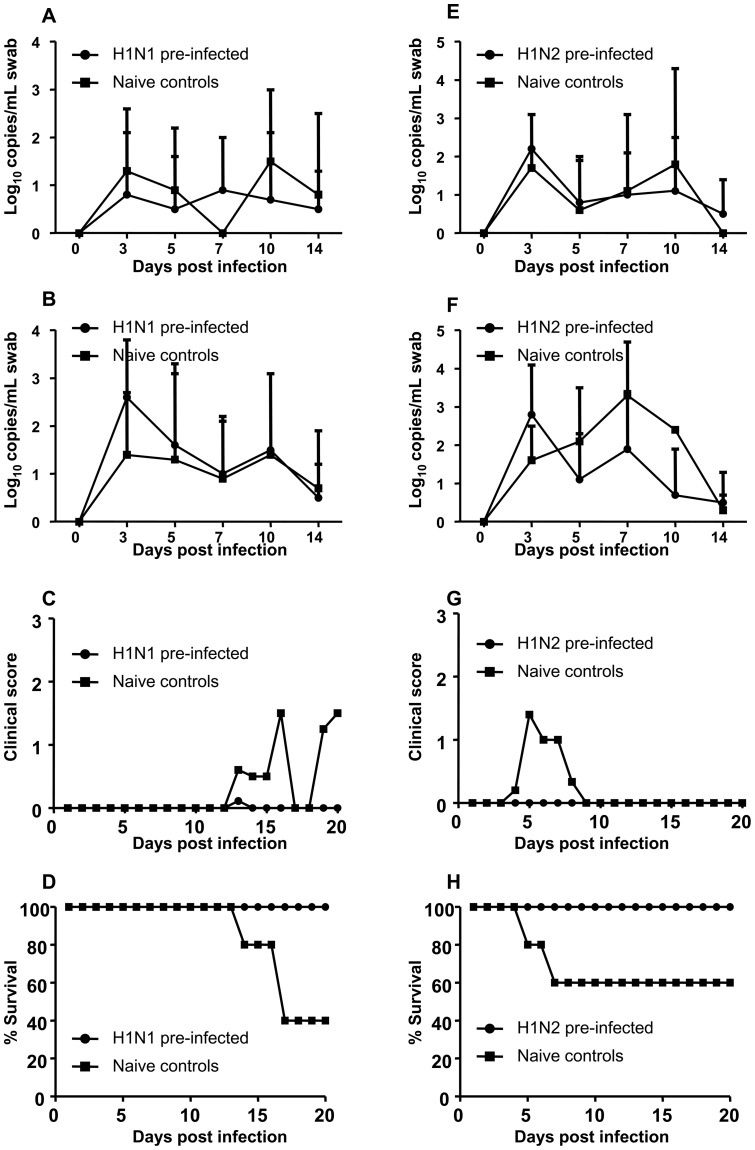
Transmission of H5N1 from H1N1 or H1N2 pre-infected chickens to naïve contact controls. Chickens previously infected with H1N1 (A–D) or H1N2 (E–H) were challenged with H5N1 and the following day contact control chickens were placed in the rooms. Virus shedding determined by quantitative real time RT-PCR in oral swabs (A and E) and cloacal swabs (B and F); clinical score (C and G) and survival of chickens (D and H) were monitored.

To evaluate if prior infection with LPAI would prevent transmission of H5N1 to contact control chickens following challenge, LPAI pre-infected chickens were challenged with H5N1 and contact control chickens were placed in the rooms the following day. H1N1 pre-infected chickens had peak viral shedding at 3 DPC which decreased with time (Figure 5A and B). Contact control chickens also shed virus starting at 3 DPC (Figure 5A and B), indicating that the pre-infected chickens shed enough virus to infect contact birds. However, virus shedding in the contact controls and the H1N1 pre-infected chickens was approximately the same at all DPC. All H1N1 pre-infected chickens did not show any clinical signs of H5N1 while the contact control chickens started to show clinical signs of disease on 13 DPC (Figure 5C). The difference in clinical score between the 2 groups was statistically significant at DPC 16, 19 and 20 (p<0.05). However, 2 contact control chickens did not develop any clinical disease at all. All H1N1 pre-infected chickens survived H5N1 challenge. On the other hand, 1 contact control chicken died on DPC 14 and 2 others on DPC 17 (Figure 5D).

Similarly, H1N2 pre-infected chickens had peak viral shedding at 3 DPC which decreased with time (Figure 5E and F). Contact control chickens also shed virus starting at 3 DPC (Figure 5E), at approximately the same levels as H1N2 pre-infected chickens in cloacal swabs at all DPCs. However, H5N1 shedding in oral swabs from contact controls was slightly higher than in H1N2 pre-infected chickens at DPC 5, 7 and 10 (Figure 5F). In addition, H1N2 pre-infected chickens did not develop clinical disease while contact control chickens started to develop clinical signs of H5N1 on 4 DPC (Figure 5G). The difference in clinical score between the 2 groups was statistically significant at DPC 5, 6 and 7 (p<0.05). All H1N2 pre-infected chickens survived H5N1 challenge (Figure 5H). However, in the contact control group, 1 chicken died on DPC 6 and another on DPC 7 (Figure 5H). Once again, 2 contact control chickens survived H5N1 challenge without showing any clinical disease.

### Pathology of chickens that died from H5N1 challenge

Histological lesions consistent with HPAI were observed in chickens that developed clinical disease. However, the organs affected and extent of the lesion was variable between individual birds examined. Common lesions included interstitial pneumonia, splenic necrosis and macrophage hyperplasia, lymphohistiocytic and necrotizing myocarditis and multifocal pancreatic necrosis. Influenza viral antigen was most consistently detected in lung, spleen, heart, skeletal muscle, kidney, proventriculus and pancreas by immunohistochemistry.

### Seroconversion in chickens following A/chicken/Vietnam/14/2005 H5N1 challenge

**Figure 6:**
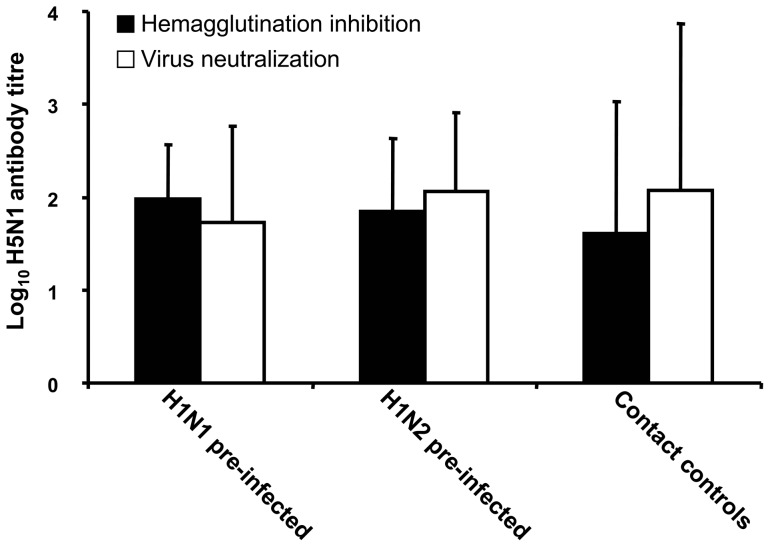
Seroconversion following H5N1 challenge. Sera from H1N1 (n = 17) or H1N2 (n = 17) pre-infected chickens and naïve contact controls (n = 4) that survived H5N1 challenge were evaluated for H5 specific antibodies by haemagglutination inhibition (HI) and virus neutralization (VN) assays. Histograms represent means of HI titres (black histograms) and VN titres (open histograms) and bars represent standard error of means.

Twenty one days following H5N1 challenge sera collected from all surviving chickens was assessed for H5 specific antibodies using an H5 competitive ELISA, H5N1 virus neutralization and HI. The majority of chickens from H1N1 and H1N2 pre-infected as well as surviving contact control chickens developed H5 specific antibodies following challenge (Figure 6).

## Discussion

In this study, the influence of prior infection with two different heterologous LPAI viruses on subsequent challenge with HPAI H5N1 was evaluated. In the first experiment, naïve control and pre-infected chickens were housed together and both challenged to allow for amplification of H5N1 following the initial challenge. Approximately 70–80% of chickens previously infected with LPAI H1N1 or H1N2 survived H5N1 challenge whereas all naïve control chickens died following acute disease. In the second experiment, only chickens previously infected with LPAI H1N1 or H1N2 were challenged with H5N1 and then naïve controls placed in the room the day following challenge to evaluate if transmission of H5N1 could occur from pre-infected chickens to naive control birds. In this second scenario the survival rate of pre-infected chickens was improved since none developed clinical disease or died from H5N1 challenge. In contrast, 40–60% of the contact control chickens died from H5N1 challenge demonstrating that the pre-infected chickens transmitted virus to the control birds. Despite transmission of H5N1 from pre-infected to naive birds, there was a delay in onset of clinical signs and a reduction in virus shedding, clinical disease and mortality. This was expected since the amount of H5N1 virus shed by chickens previously infected with either H1N1 or H1N2 was two orders of magnitude lower then the H5N1 challenge dose used. This study demonstrates that previous infection with a LPAI H1N1 or H1N2 was able to partially protect chickens against HPAI H5N1 challenge.

This heterologous protection is not mediated by neutralizing antibodies since antibodies to H5 were not detectable prior to challenge. However, antibodies developed against both H1N1 and H1N2 infection in chickens were able to decrease H5N1 virus plaque size compared to naïve serum. This inhibition of virus spread in the absence of specific neutralizing activity indicates that non-neutralizing antibodies may play a role in the clearance of H5N1. Antibodies against the NA protein can prevent the release of new virus particles from infected cells thereby restricting virus replication [21], [33]. Therefore, the anti N1 antibodies in chickens pre-infected with H1N1 could have potentially restricted H5N1 replication, consequently reducing disease and mortality. The H5N1 plaque size reduction was slightly better using H1N1 sera compare to H1N2 sera however this was not statistically significant. However, it is likely that the H1N1 sera N1 component is responsible for this observation as sera from H1N1 infected chickens developed N1 specific antibodies determined using a neuraminidase inhibition assay. Similarly, this could have been the case in H1N2 infected chickens if anti-N2 antibodies were to cross-react with the N1 of H5N1. However, this is very unlikely given that there was only 43% homology between the NA proteins of H1N2 and H5N1, with a 20 amino acid deletion in the stalk region of N1 from H5N1. Together this indicates that N1 antibodies as well as other antibodies may be responsible for H5N1 plaque size reduction. Therefore, additional mechanisms were likely involved in the protection against H5N1. Antibodies against the matrix protein 2 (M2) would prevent the uncoating of influenza virus during infection and thus restrict virus replication. This has been demonstrated using monoclonal antibodies against influenza M2 which reduced influenza virus plaque size without affecting the number of plaques [31]. In addition, this antibody reduced the replication of influenza virus in mouse lungs, indicating that antibodies against M2 can provide protection in vivo [34].

However, heterologous cell mediated immunity against influenza generated at mucosal sites is likely another mechanism responsible for protection. Prior infection of chickens with H9N2 was able to protect them from H5N1 and this protection was cell mediated because adoptive transfer of cytotoxic CD8 T cells from H9N2 primed chickens protected naïve inbred chickens from H5N1 challenge [26]. A similar mechanism is responsible for protection against H5N1 in mice previously infected with H9N2 [35] and pigs previously infected with swine influenza H1N1 [21]. The highly conserved internal proteins (PB2, PB1, PA and NP) of influenza virus are the most likely source of cross-reactive epitopes recognised by cytotoxic T cells [36], [37].

The immunity generated by prior infection with LPAI H1N1 or H1N2 was not able to prevent virus replication; however it did decrease shedding of HPAI H5N1 in oral and cloacal swabs. Reduced virus shedding is expected in this situation since infection can not be completely prevented in the absence of neutralizing antibodies. Prior infection with H9N2 was able to protect chickens from H5N1 but surviving birds shed low amounts of virus in feces [26]. Similarly, when Canada geese are pre-exposed to LPAI they develop partial protection against HPAI [22]. In addition, in wood ducks, prior infection with H1N1 influenza provided partial protection against H5N1 [23].

Chickens that survived H5N1 challenge generated antibodies specific for H5. This seroconversion for H5 allows for the differentiation of infected and vaccinated animals (DIVA), whereby H1 specific antibodies are only present in chickens pre-infected with H1N1 or H1N2. Vaccination of chickens using LPAI to protect against HPAI H5, with diagnostic tests for DIVA could be used for improved control of HPAI in endemic countries. In addition, it could be most effective when all chickens are vaccinated to generate herd immunity as 100% protection was demonstrated when only H1N1 or H1N2 pre-infected chickens were challenged compared to 70–80% protection where pre-infected chickens were mixed with naïve chickens and all challenged with H5N1. An ideal vaccine should prevent virus infection and shedding via neutralizing antibodies. Therefore, partial protection provided by LPAI against H5N1 is potentially dangerous as it can mask the clinical manifestation of H5N1 allowing the virus to spread. Nevertheless, a reduction in virus shedding, severity of illness and deaths due to IAV might be acceptable outcomes during a pandemic. This can then be combined with a DIVA to identify and eliminate H5-positive chickens. The duration of immunity is unknown; however, it is likely that it would last for the usually short lifetime of a chicken. A major question remains if a similar strategy of using a low pathogenic influenza virus in humans that does not cause disease would elicit protective immunity against H5N1. Epidemiological data from Vietnam suggests that previous and probably repeated infection of humans by influenza makes them less likely to die from H5N1 infection. This was based largely on the finding that humans aged ≤16 years were more likely to die from H5N1 infection than did older people [38]. In humans, protection against influenza H3N2 or H1N1 challenge correlated with pre-existing influenza-specific CD4+ T cells [39]. It has been previously demonstrated that prior infection with H3N2 in ferrets can protect against H5N1 illustrating that protection against H5N1 is possible in other animal species. Furthermore, using a temperature sensitive attenuated H1N1 influenza virus, it was recently demonstrated that protection against H5N1 could be achieved in mice [40]. It is possible that the protective immunity generated by replicating low pathogenic influenza viruses may be different between different animal species including humans.

Chickens are one of the most susceptible species to HPAI H5N1 with mortalities approaching 100%. We have demonstrated in chickens that protective immunity can be generated in the absence of H5 specific neutralizing antibodies. We are currently investigating if live attenuated influenza vaccines can elicit similar protection as the LPAI against HPAI in chickens. The development of several live attenuated influenza viruses covering the HA subtypes that have a pandemic potential would enhance influenza pandemic preparedness. Such vaccines can be manufactured and stockpiled prior to the emergence of a new influenza virus and then immediately tested for efficacy, thereby avoiding the need to develop and produce a new vaccine at the beginning of an outbreak.

## References

[1] OIE (2008) Office International des Epizooties Manual of Diagnostic Tests and Vaccines for Terrestrial Animals (mammals, birds and bees): 465–481. 16642778

[2] Pasick J, Handel K, Robinson J, Copps J, Ridd D, et al (2005) Intersegmental recombination between the haemagglutinin and matrix genes was responsible for the emergence of a highly pathogenic H7N3 avian influenza virus in British Columbia. J Gen Virol 86: 727–731. 15722533 10.1099/vir.0.80478-0

[3] Suarez DL, Senne DA (2000) Sequence analysis of related low-pathogenic and highly pathogenic H5N2 avian influenza isolates from United States live bird markets and poultry farms from 1983 to 1989. Avian Dis 44: 356–364. 10879916

[4] Suarez DL, Perdue ML, Cox N, Rowe T, Bender C, et al (1998) Comparisons of highly virulent H5N1 influenza A viruses isolated from humans and chickens from Hong Kong. J Virol 72: 6678–6688. 9658115 10.1128/jvi.72.8.6678-6688.1998 PMC109865

[5] Shortridge KF, Zhou NN, Guan Y, Gao P, Ito T, et al (1998) Characterization of avian H5N1 influenza viruses from poultry in Hong Kong. Virology 252: 331–342. 9878612 10.1006/viro.1998.9488

[6] Claas EC, de Jong JC, van Beek R, Rimmelzwaan GF, Osterhaus AD (1998) Human influenza virus A/HongKong/156/97 (H5N1) infection. Vaccine 16: 977–978. 9682346 10.1016/s0264-410x(98)00005-x

[7] Buxton Bridges C, Katz JM, Seto WH, Chan PK, Tsang D, et al (2000) Risk of influenza A (H5N1) infection among health care workers exposed to patients with influenza A (H5N1), Hong Kong. J Infect Dis 181: 344–348. 10608786 10.1086/315213

[8] Kawaoka Y (2012) H5N1: Flu transmission work is urgent. Nature 482: 155. 22278057 10.1038/nature10884

[9] Fouchier RA, Herfst S, Osterhaus AD (2012) Public health and biosecurity. Restricted data on influenza H5N1 virus transmission. Science 335: 662–663. 22267582 10.1126/science.1218376

[10] Webster RG, Bean WJ, Gorman OT, Chambers TM, Kawaoka Y (1992) Evolution and ecology of influenza A viruses. Microbiol Rev 56: 152–179. 1579108 10.1128/mr.56.1.152-179.1992 PMC372859

[11] Olsen B, Munster VJ, Wallensten A, Waldenstrom J, Osterhaus AD, et al (2006) Global patterns of influenza a virus in wild birds. Science 312: 384–388. 16627734 10.1126/science.1122438

[12] Tong S, Li Y, Rivailler P, Conrardy C, Castillo DA, et al (2012) A distinct lineage of influenza A virus from bats. Proc Natl Acad Sci U S A 109: 4269–4274. 22371588 10.1073/pnas.1116200109 PMC3306675

[13] Butler D (2010) Portrait of a year-old pandemic. Nature 464: 1112–1113. 20414281 10.1038/4641112a

[14] Lee CW, Senne DA, Suarez DL (2004) Effect of vaccine use in the evolution of Mexican lineage H5N2 avian influenza virus. J Virol 78: 8372–8381. 15254209 10.1128/JVI.78.15.8372-8381.2004 PMC446090

[15] Grund C, Abdelwhab el SM, Arafa AS, Ziller M, Hassan MK, et al (2011) Highly pathogenic avian influenza virus H5N1 from Egypt escapes vaccine-induced immunity but confers clinical protection against a heterologous clade 2.2.1 Egyptian isolate. Vaccine 29: 5567–5573. 21244859 10.1016/j.vaccine.2011.01.006

[16] Patel A, Tran K, Gray M, Li Y, Ao Z, et al (2009) Evaluation of conserved and variable influenza antigens for immunization against different isolates of H5N1 viruses. Vaccine 27: 3083–3089. 19428922 10.1016/j.vaccine.2009.03.023

[17] Gao W, Soloff AC, Lu X, Montecalvo A, Nguyen DC, et al (2006) Protection of mice and poultry from lethal H5N1 avian influenza virus through adenovirus-based immunization. J Virol 80: 1959–1964. 16439551 10.1128/JVI.80.4.1959-1964.2006 PMC1367171

[18] Masic A, Booth JS, Mutwiri GK, Babiuk LA, Zhou Y (2009) Elastase-dependent live attenuated swine influenza A viruses are immunogenic and confer protection against swine influenza A virus infection in pigs. J Virol 83: 10198–10210. 19625412 10.1128/JVI.00926-09 PMC2748057

[19] Grebe KM, Yewdell JW, Bennink JR (2008) Heterosubtypic immunity to influenza A virus: where do we stand? Microbes Infect 10: 1024–1029. 18662798 10.1016/j.micinf.2008.07.002 PMC2584237

[20] Bodewes R, Kreijtz JH, Geelhoed-Mieras MM, van Amerongen G, Verburgh RJ, et al (2011) Vaccination against seasonal influenza A/H3N2 virus reduces the induction of heterosubtypic immunity against influenza A/H5N1 virus infection in ferrets. J Virol 85: 2695–2702. 21228239 10.1128/JVI.02371-10 PMC3067975

[21] Van Reeth K, Braeckmans D, Cox E, Van Borm S, van den Berg T, et al (2009) Prior infection with an H1N1 swine influenza virus partially protects pigs against a low pathogenic H5N1 avian influenza virus. Vaccine 27: 6330–6339. 19840669 10.1016/j.vaccine.2009.03.021

[22] Berhane Y, Leith M, Embury-Hyatt C, Neufeld J, Babiuk S, et al (2010) Studying possible cross-protection of Canada geese preexposed to North American low pathogenicity avian influenza virus strains (H3N8, H4N6, and H5N2) against an H5N1 highly pathogenic avian influenza challenge. Avian Dis 54: 548–554. 20521692 10.1637/8841-040309-Reg.1

[23] Costa TP, Brown JD, Howerth EW, Stallknecht DE, Swayne DE (2011) Homo- and heterosubtypic low pathogenic avian influenza exposure on H5N1 highly pathogenic avian influenza virus infection in wood ducks (Aix sponsa). PLoS One 6: e15987. 21253608 10.1371/journal.pone.0015987 PMC3017094

[24] Fereidouni SR, Starick E, Beer M, Wilking H, Kalthoff D, et al (2009) Highly pathogenic avian influenza virus infection of mallards with homo- and heterosubtypic immunity induced by low pathogenic avian influenza viruses. PLoS One 4: e6706. 19693268 10.1371/journal.pone.0006706 PMC2724736

[25] Kalthoff D, Breithaupt A, Teifke JP, Globig A, Harder T, et al (2008) Highly pathogenic avian influenza virus (H5N1) in experimentally infected adult mute swans. Emerg Infect Dis 14: 1267–1270. 18680652 10.3201/eid1408.080078 PMC2600380

[26] Seo SH, Webster RG (2001) Cross-reactive, cell-mediated immunity and protection of chickens from lethal H5N1 influenza virus infection in Hong Kong poultry markets. J Virol 75: 2516–2525. 11222674 10.1128/JVI.75.6.2516-2525.2001 PMC115873

[27] Spackman E, Senne DA, Myers TJ, Bulaga LL, Garber LP, et al (2002) Development of a real-time reverse transcriptase PCR assay for type A influenza virus and the avian H5 and H7 hemagglutinin subtypes. J Clin Microbiol 40: 3256–3260. 12202562 10.1128/JCM.40.9.3256-3260.2002 PMC130722

[28] Webster R CN, Stohr K. (2002) World Health Organization Manual on Animal Influenza Diagnosis and Surveillance. WHO.

[29] Pederson J (2008) Avian Influenza A Viruses. Methods in molecular biology. 67–75.

[30] Zhou EM, Chan M, Heckert RA, Riva J, Cantin MF (1998) Evaluation of a competitive ELISA for detection of antibodies against avian influenza virus nucleoprotein. Avian Dis 42: 517–522. 9777152

[31] Zebedee SL, Lamb RA (1988) Influenza A virus M2 protein: monoclonal antibody restriction of virus growth and detection of M2 in virions. J Virol 62: 2762–2772. 2455818 10.1128/jvi.62.8.2762-2772.1988 PMC253710

[32] Dalgaard TS, Norup LR, Pedersen AR, Handberg KJ, Jorgensen PH, et al (2010) Flow cytometric assessment of chicken T cell-mediated immune responses after Newcastle disease virus vaccination and challenge. Vaccine 28: 4506–4514. 20434546 10.1016/j.vaccine.2010.04.044

[33] Sandbulte MR, Jimenez GS, Boon AC, Smith LR, Treanor JJ, et al (2007) Cross-reactive neuraminidase antibodies afford partial protection against H5N1 in mice and are present in unexposed humans. PLoS Med 4: e59. 17298168 10.1371/journal.pmed.0040059 PMC1796909

[34] Treanor JJ, Tierney EL, Zebedee SL, Lamb RA, Murphy BR (1990) Passively transferred monoclonal antibody to the M2 protein inhibits influenza A virus replication in mice. J Virol 64: 1375–1377. 2304147 10.1128/jvi.64.3.1375-1377.1990 PMC249260

[35] O'Neill E, Krauss SL, Riberdy JM, Webster RG, Woodland DL (2000) Heterologous protection against lethal A/HongKong/156/97 (H5N1) influenza virus infection in C57BL/6 mice. J Gen Virol 81: 2689–2696. 11038381 10.1099/0022-1317-81-11-2689

[36] Bennink JR, Yewdell JW, Smith GL, Moss B (1987) Anti-influenza virus cytotoxic T lymphocytes recognize the three viral polymerases and a nonstructural protein: responsiveness to individual viral antigens is major histocompatibility complex controlled. J Virol 61: 1098–1102. 3493353 10.1128/jvi.61.4.1098-1102.1987 PMC254069

[37] Subbarao K, Joseph T (2007) Scientific barriers to developing vaccines against avian influenza viruses. Nat Rev Immunol 7: 267–278. 17363960 10.1038/nri2054 PMC7097526

[38] Liem NT, Tung CV, Hien ND, Hien TT, Chau NQ, et al (2009) Clinical features of human influenza A (H5N1) infection in Vietnam: 2004–2006. Clin Infect Dis 48: 1639–1646. 19435433 10.1086/599031 PMC7116471

[39] Wilkinson TM, Li CK, Chui CS, Huang AK, Perkins M, et al (2012) Preexisting influenza-specific CD4+ T cells correlate with disease protection against influenza challenge in humans. Nat Med 18: 274–280. 22286307 10.1038/nm.2612

[40] Shi J, Wen Z, Guo J, Zhang Y, Deng G, et al (2012) Protective efficacy of an H1N1 cold-adapted live vaccine against the 2009 pandemic H1N1, seasonal H1N1, and H5N1 influenza viruses in mice. Antiviral Res 93: 346–353. 22281419 10.1016/j.antiviral.2012.01.001

